# Mechanism of anti-biofilm thymol activity in oral *Candida albicans* strains – phenotypic and genotypic level

**DOI:** 10.1080/20002297.2026.2741862

**Published:** 2026-10-29

**Authors:** Anna Biernasiuk, Marta Ostrowska-Leśko, Anna Malm, Agnieszka Korga-Plewko

**Affiliations:** a Department of Pharmaceutical Microbiology, Faculty of Pharmacy, Medical University of Lublin, Lublin, Poland; b Independent Laboratory of Translational Medicine, Chair of Medical Genetics, Medical University of Lublin, Lublin, Poland; c Independent Medical Biology Unit, Faculty of Pharmacy, Medical University of Lublin, Lublin, Poland

**Keywords:** Thymol, *Candida albicans*, biofilm, mode of action, filamentation, gene expression

## Abstract

**Background:**

Recently, the incidences of superficial infections caused by *Candida albicans* has been increasing. In turn, the limited number of available antimycotics has led to the search for new antifungals.

**Objective:**

The purpose of this study was to evaluate the antifungal activity, especially the anti-biofilm effect, of thymol (THY) towards oral *C. albicans* isolates from hemato-oncological patients.

**Design:**

The studies included: (1) the evaluation of the minimum inhibitory concentration and minimum fungicidal concentration of THY against *C. albicans* as found using the broth microdilution method; (2) its effect on biofilm inhibition and reduction, as assessed by way of a similar technique with crystal violet staining; (3) the interactions of THY with yeast cellular structures, as brought to light via sorbitol and ergosterol tests; (4) its influence on the filamentation as revealed through the use of an optical microscope and (5) the impact on the expression of selected biofilm genes, as determined by quantitative real-time PCR.

**Results:**

THY showed good or moderate anticandidal activity with fungicidal effect. The concentrations required to inhibit the formation or eradication of mature biofilm were higher. These results suggest that THY may likely have an effect on the cell membrane and filamentation at *C. albicans.* Exploratory RT-qPCR analysis indicated a directional increase in *BCR1* expression under biofilm-forming conditions (unadjusted *p* = 0.010), but this result did not remain statistically significant after Benjamini‒Hochberg correction (*q* = 0.080). *ALS3, HWP1* and *NRG1* showed non-significant directional trends, and no gene revealed a statistically significant change under planktonic conditions.

**Conclusion:**

THY demonstrated a multidirectional mechanism of anticandidal action, which may suggest its promising effect in the prevention and treatment of oral infections.

## Key messages

1) Thymol showed multidirectional effect against *C. albicans* with fungicidal and antibiofilm activity.
2) Thymol probably acts at the level of the fungal membrane by interfering with ergosterol, and also inhibits the filamentation process, which may decrease the adhesion of *C. albicans* to surfaces.
3) Exploratory RT-qPCR data showed heterogeneous, isolate-dependent responses to thymol. *BCR1* had the least unadjusted *p*-value in biofilm-associated cells, but no gene remained statistically significant after multiple-testing correction; the other directional changes should be regarded as non-significant trends.

## Graphical abstract

**Figure:**
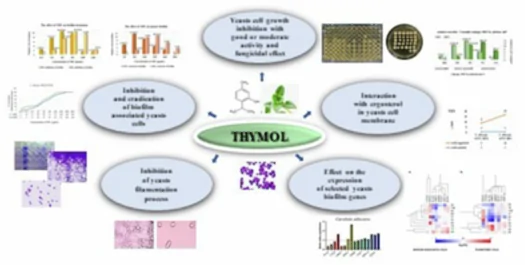

## Introduction

Diseases caused by fungi can be considered a major medical problem worldwide. These microorganisms are responsible for more than 150 million severe cases of fungal infections and an annual death toll approaching 2 million [1,2]. Factors that predispose to these infections include primarily a weakened immune system, diabetes, solid organ and hematologic malignancies cancers, organ transplants, immunosuppression or antibiotic and steroid therapy [1–6]. The most common opportunistic infections are those caused by yeasts from the human microbiota belonging to the *Candida* species. These fungi can cause superficial, invasive and even fatal candidiasis [3–5,7,8]. The most medically important is *C. albicans,* which colonizes the oral mucosal surfaces of 30–50% of all healthy people. The rate of carriage increases with age and in persons with dental prostheses by up to 60% [9]. Candidiasis can affect the oral cavity [7,10], skin, gastrointestinal and urogenital tract, lungs and other parts of the body [5,6,9]. *C. albicans* accounts for approximately 75–80% of all these infections [1,9]. WHO data also shows that over the past 10 years, one of the most common etiological factors of nosocomial infections has been the yeast *C. albicans* (in 15% of all patients) [3].

The main virulence factors of *Candida* spp. include the ability to adhere to host tissues and biofilm formation, the transition of yeast to hyphae, phenotypic switching, subsequent metabolic adaptation, the production of invasins and the secretion of hydrolases [3,9,10]. Most *Candida* infections are particularly associated with biofilm formation. This specific community of microorganisms forms on biotic (mucous membranes) or abiotic surfaces (dentures, catheters or other implanted medical devices). It is surrounded by an extracellular matrix of exopolysaccharides (EPS) [3,7,8]. Therefore, *C. albicans* may protect itself from host immune attacks and tolerate higher concentrations of antifungal agents [6].

Currently, only a few classes of antifungal agents are available and can be used for the treatment of infections caused by *Candida* spp. These are: polyenes (e.g. amphotericin B or nystatin), azoles (e.g. fluconazole or posaconazole) and echinocandins (e.g. caspofungin or micafungin) [11]. The scarcity of antifungal drugs, rapid emergence of resistance to antimycotics, their long-term use and the severity of adverse effects eliminates effective treatment options for candidiasis [5,8,12]. Furthermore, there are a limited number of unique drug targets in fungal cells [2]. This situation has intensified interest in research on new antifungal compounds, including natural substances, that could be used in alternative therapies such as antimycotics or antiseptics.

Topically applied antiseptics (skin or mouthwashes), notably, may represent suitable tools for fungal infection control and local therapy [12–14]. These may contain selected components with different mechanisms of action, and many have known antibacterial activity, while antifungal effect sometimes remains unclear [13,14]. Recently, however, numerous studies have underscored the antimicrobial effectiveness of natural products. Hence, herbal agents can be considered potential tools for preventing and managing infectious oral diseases [8]. Their contained phytochemicals, among others, extracts and plant derivatives, essential oils and their components, such as thymol (THY), eugenol and linalool, are particularly promising antifungal agents [1,11].

THY, also known as 2-isopropyl-5-methylphenol, is an isomeric phenolic monoterpene with a strong odor and good solubility in alcohol and other organic solvents but is poorly water-soluble. It occurs abundantly in various concentration ranges of 30–80% in the essential oils (EOs) of certain plants, such as *Thymus vulgaris, Thymus zygis, Thymus ciliates, Thymus pectinatus, Satureja hortensis, Satureja intermedia, Satureja thymbra, Thymbra capitata, Ocimum gratissimum, Trachyspermum ammi, Carum copticum, Lippia multiflora, Zataria multiflora, Centipeda minima* or *Nigella sativa*[15,16]. THY exhibits a wide range of biological activities and has been used in medicine for many years due to its different pharmacological properties. These include applications such as antirheumatics, antioxidants, free radical scavengers, analgesics, antispasmodics, anti-inflammatories, anticancer agents, antiseptics and antimicrobials [15–19]. THY is generally regarded as safe (GRAS—generally recognized as safe) by the Food and Drug Administration (FDA) for human consumption and food additives [15,17]. It is often employed for oral hygiene and dentistry applications as an ingredient of mouthwashes, toothpastes and antiseptic solutions. Moreover, it is approved as food flavorings and has been considered as an antibacterial additive in food and feed [17]. It is one of the potential candidates of natural origin that has shown promising therapeutic properties and pharmacological or pharmacokinetic effects for pharmaceutical development [15]. THY is known for its antimicrobial properties both *in vitro* and *in vivo,* but some of its activities have not yet been thoroughly investigated [17,20]. Still, investigations into its application as an antifungal must recognize that antifungal resistance is inevitable. Hence, it is crucial to obtain insight into the molecular mechanisms that govern the tolerance of THY so as to gain an understanding of specific targets in developing novel and effective therapeutic strategies [17].

Therefore, the purpose of the present work was to evaluate *in vitro* the antifungal activity of THY towards reference and clinical strains of *C. albicans* derived from the oral cavity of hospitalized patients with hematological malignancies. The antibiofilm activity of THY and its mechanisms of action, including interactions with fungal cell structures and its influence on the filamentation process, were assessed. Additionally, the effect of THY on the expression of selected genes involved in biofilm formation was analyzed.

## Materials and methods

### The studied compounds

In our research, THY and nystatin (as control) (Sigma-Aldrich Chemicals, St. Louis, MO, USA) were used. Their activity was determined *in vitro* against the yeast *C. albicans*. Dimethyl sulfoxide—DMSO (Pol-Aura, Różnowo, Poland) was employed to dissolve these substances and obtain a stock solution for testing.

### Microorganisms

The research was carried out upon fungi of the *C. albicans* species. The yeasts belonged to both reference material from the American Type Culture Collection (ATCC) and clinical strains. Among the reference strains, *Candida albicans* ATCC 2091 and *Candida albicans* ATCC 10231 were used. In turn, thirty clinical strains were obtained from the oral candidiasis of patients hospitalized for hematological malignancies. The study protocol was approved by the Ethics Committee of the Medical University of Lublin (no. KE-0254/75/2011). The isolates were identified by standard diagnostic tests: microscopic, macroscopic and a biochemical method based on the growth on chromogenic agar (Brilliance™ Candida Agar, (Argenta, Poznań, Poland)), as well as assimilation of different substrates using the VITEK 2 automatic system with ID-YST cards (bioMèrieux S.A., Warsaw, Poland). The strains were stored as glycerol stock at −70 °C. The yeasts were cultured for testing at 37 °C for 24 h on Sabouraud agar (BioMaxima S.A., Lublin, Poland).

### Assessment of THY antifungal activity

The activity of THY towards *C. albicans* was examined using the broth microdilution method via 96-well polystyrene plates, according to the recommendations of the European Committee on Antimicrobial Susceptibility Testing (EUCAST) [21] and the Clinical and Laboratory Standards Institute (CLSI) [22]. The studies were conducted as described earlier [11]. First, a stock solution of THY in DMSO was prepared at a concentration of 50 mg/mL. The minimum inhibitory concentration (MIC) of THY was then tested by two-fold dilutions in RPMI (Roswell Park Memorial Institute) 1640 broth supplemented with MOPS (3-(*N*-morpholino) propanesulfonic acid) (Sigma-Aldrich Chemicals, St. Louis, MO, USA). The range of final THY concentrations was 3.91–8,000 μg/mL. After culturing the yeasts on Sabouraud dextrose agar (SDA) (37 °C, 24 h), they were suspended in sterile 0.85% NaCl with an optical density on the McFarland scale of 0.5—approximately 5 × 10^5^ CFU/mL (Colony Forming Units/mL). Next, 1 μL of each fungal suspension was added to wells containing 100 μL of RPMI 1640 broth with MOPS and various concentrations of THY. After incubation (under the same conditions as before), the MIC was determined using a spectrophotometer (as the lowest THY concentration that showed complete inhibition of fungal growth). DMSO, growth and sterility controls were performed in parallel. Nystatin (NYS) (Sigma-Aldrich, Chemicals, St. Louis, MO, USA) served as a positive control. In the following stage, the minimum fungicidal concentration (MFC) of THY was established (as the lowest concentration causing the killing of fungal strains). The MFC assay was performed by transferring individual cultures from each well of the titration plate to SDA medium and re-incubating. The lowest concentrations of THY causing no growth of *C. albicans* were considered fungicidal. These studies were performed 3 times, and representative results are presented here [11].

In this study, mild bioactivity of THY was defined as a MIC in the range 501–1,000 μg/mL, moderate bioactivity, with MIC from 126 to 500 μg/mL, good bioactivity as a MIC in the range 26–125 μg/mL [23]. The fungicidal (MFC/MIC ≤ 4) or fungistatic (MFC/MIC > 4) effect of this terpene was also assessed [23,24].

### Evaluation of *C. albicans* biofilm formation

Biofilm formation was assessed *in vitro* using 96-well polystyrene microplates (Thermo Scientific™ Nunc™, Denmark) by way of crystal violet (CV) staining (Sigma–Aldrich, USA). The quantification of total biofilm biomass included both living cells, dead cells and matrix material. The isolates of *C. albicans* were inoculated on Sabouraud's dextrose broth (SDB) and incubated at 37 °C for 24 h. Cell suspensions of 1 × 10^6^ cells/mL were then prepared in SDB. Afterwards, 100 μL of cell suspensions was added to selected wells of commercially available pre-sterilized, flat-bottomed, 96-well polystyrene microtiter plates (Thermo Scientific™ Nunc™ Brank Product, Roskilde, Denmark) and incubated at 37 °C for 48 h in order to obtain mature biofilms. Once the medium above the culture was decanted, non-adherent or loosely adherent cells were removed by thoroughly washing 3 times in 100 μL sterile phosphate buffer solution (PBS). Subsequently, the plates were dried in an inverted position and then stained with 100 μL of 0.1% CV for 15 min. Following this, the plates were washed under distilled water to remove unbound dye. Next, in order to elicit a response to each of the wells, 100 μL of isopropyl alcohol (bioMerieux) was added, and the plates were left at room temperature for 15 min to solubilize the CV. The medium was used as the blank control. The optical density (OD) of the alcohol-dye solution in each well was read at wave length *λ* = 600 (OD_600_) by means of a microplate reader (BioTek ELx800) [25–29].

The cut-off optic density (OD_c_) for the microtiter-plate test was defined as three standard deviations (SD) above the mean OD of the negative control and was calculated separately for each microtiter plate. Based on these values, the ability of *C. albicans* cells to form a biofilm on the surface of a microtiter plate was assessed and described according to the following classification: OD ≤ ODc = biofilm nonproducer, ODc < OD ≤ 2 × ODc = weak biofilm producer, 2 × ODc < OD ≤ 4 × ODc = moderate biofilm producer and 4 × ODc < OD = strong biofilm producer [27,28].

### Evaluation of *C. albicans* biofilm biomass inhibition by THY

The effect of THY on biofilm formation of *C. albicans* was examined according to the protocol previously described [25,26] by exercising the *in vitro* microdilution method upon a stationary culture, and employing flat-bottomed, 96-well polystyrene microplates (Thermo Scientific™ Nunc™, Denmark) and crystal violet (CV) staining (Sigma–Aldrich, USA). The quantification of total biofilm biomass included both living cells, dead cells and matrix material. Briefly, cell suspensions in RPMI 1640 medium at a density of 10^6^ CFU/mL were created from colonies of each representative *C. albicans* strain subcultured on SDA (37 °C, 24 h). Serial two-fold diluted concentrations of THY (in the range 15.62–8,000 μg/mL) were then made up on microtiter plates at 100 μL volumes. The same volume of the prepared *C. albicans* suspension was subsequently added to the wells and mixed with THY. After incubation (37 °C, 24 h), the supernatants were discarded, and the microtiter plates were washed 3 times with 100 μL PBS. The plates were then dried in the inverted position and stained with 100 μL of 0.1% CV for 15 min to stain the cells. The plates were washed under distilled water to remove unbound dye. Next, in order to elicit a response to each of the wells, 100 μL of isopropyl alcohol (bioMerieux) was added, and the plates were left at room temperature for 15 min to solubilize the CV. THY in the same concentrations, PBS and the medium alone were used as the positive, non-treated and blank controls, respectively. The optical density (OD) of the alcohol-dye solution in each well was read at wave length *λ* = 600 (OD_600_) by means of a microplate reader (BioTek ELx800). Herein, higher OD values indicate an increased number of *C. albicans* in the biofilm. The percentage inhibition was calculated using the equation: [1 − (A600 of the test/A600 of the non-treated control)] × 100%. All the experiments were performed in triplicate. Our study also included microscopic visualization of the adherence and biofilm forming of *C. albicans* cells [25–29].

### Investigation of *C. albicans* biofilm biomass reduction by THY

This study was performed through the use of the microdilution method (similarly to the evaluation of biofilm biomass inhibition, but with some differences) [25,26]. Cell suspensions in RPMI 1640 medium at a density of 10^6^ CFU/mL were prepared from *C. albicans* colonies subcultured on SDA (37 °C, 24 h). After this, 100 μL of each representative *C. albicans* suspensions was inoculated into microtiter plate wells and incubated as above. Following biofilm formation, the supernatants were discarded, and the microtiter plate wells were carefully washed 3 times with 100 μL PBS in order to remove non-adherent cells. THY was then added to the mature biofilms in two-fold serial dilutions (as above), and the microplates were re-incubated. After incubation, the wells were again washed 3 times with sterile PBS. Further testing was performed in the same manner as for the evaluation of biofilm biomass inhibition. The percentage reduction was ascertained by way of applying the equation: [1 − (A600 of the test/A600 of the nontreated control)] × 100%. All the experiments were performed in triplicate [25–29].

### Mechanism of antifungal action of THY

#### Sorbitol assay

The sorbitol test was applied to discern the action of THY on *C. albicans* cell walls [30–33]. This study was carried out by way of the microdilution method, similar to the MIC assessment of this natural phytocomponent. The MIC of THY against the various yeasts was re-determined simultaneously using Sabouraud dextrose broth (SDB) medium (BioMaxima S.A., Lublin, Poland) in the presence and absence of 0.8 M sorbitol (Sigma‒Aldrich Chemicals, St. Louis, MO, USA). First, 100 μL of SDB with or without sorbitol was added to the microplate wells, followed by serial dilutions of THY (3.91–8,000 μg/mL) and NYS (0.004–1,000 μg/mL) for comparison. After introducing 10 μL of *C. albicans* suspension to each well, the plates were incubated. The MIC values of both substances were assessed after 2 and 7 days, and yeast growth and sterility were checked. These studies were performed 3 times (as before) [30–33].

#### Ergosterol assay

The ergosterol test was administered to determine the mechanism of action of THY on *C. albicans* cytoplasmic membrane and its sterols [30–33]. The microdilution method was applied for the study, as before. In parallel, the MIC of THY was assessed using SDB medium with and without exogenous ergosterol (Sigma–Aldrich Chemicals, St. Louis, MO, USA). The initial concentration of the ergosterol solution was 10 mg/mL, and the final concentration in the medium was 400 μg/mL. After the addition of 100 μL of SDB medium with and without ergosterol to all the microplate wells, two-fold dilutions of THY and NYS (as control) were undertaken. Herein, the concentration ranges of both substances were the same (as in the sorbitol test: 3.91–8,000 μg/mL and 0.004–1,000 μg/mL, respectively). As before, 10 μL of yeast suspension was transferred to all wells. The microplates were then incubated (37 °C, 24 h), and the MIC values were read. Yeast growth and sterility were determined. These studies were also performed as 3 replicates (as before) [30–33].

### Evaluation of the effect of THY on the filamentation process of *C. albicans* cells

Reference strains of *C. albicans* were incubated in SDB medium for 18–24 h at 37 °C. The cultures were then centrifuged and suspended to obtain 0.6 OD (1.8 McFarland scale) in fresh SDB (700 μL) with human serum (300 μL). THY was added at their final concentration of 1/4 MIC, 1/2 MIC and MIC, and the cells were incubated for 24 h at 37 °C. The cells incubated without THY were used as control. Filament formation was analyzed in bright field (40× objective), using an Olympus BX51 microscope with a DP25 camera (Olympus, Japan Tokyo) [34].

### Assessment of the effect of THY on the expression of genes responsible for *C. albicans* adhesion and biofilm formation

#### mRNA expression analysis

Gene expression studies involved ten strong biofilm-producing *C. albicans* clinical isolates. The presence of selected adhesion and biofilm-related genes (*ALS3, CYS3, HWP1, NRG1, SAM2, TUP1, GRF10, BCR1*) had been previously confirmed in these strains, and the effect of THY at a concentration of 62.5 μg/mL on the expression of these genes was evaluated. This concentration was selected as the lowest THY concentration producing measurable biological effects in preliminary phenotypic assays. Although this concentration was below the MIC for most isolates, it corresponded to the MIC value for a subset of strains and therefore should be interpreted as a low-exposure rather than a universally sub-inhibitory concentration.

Gene expression data were visualized using heatmap analysis based on log2-transformed relative expression values log_2_(RQ) obtained via the ΔΔCt method. Hierarchical clustering was performed through one-minus Pearson correlation as the distance metric and average linkage via Morpheus software (https://software.broadinstitute.org/morpheus/). Here, rows represented individual clinical isolates, and columns represented the analyzed biofilm-associated genes. The color scale was centered at zero, indicating no change in expression relative to the untreated control.

#### RNA extraction and cDNA synthesis

Total RNA was isolated from 1 mL of fresh yeast cultures, both untreated and treated with THY. Prior to extraction, enzymatic cell lysis was performed with 200U of lyticase (A&A Biotechnology, Poland). RNA was then purified by employing the Syngen Tissue RNA Mini Kit (Syngen Biotech, Poland) according to the manufacturer's protocol. The residual genomic DNA was digested with amplification-grade DNase I (Sigma-Aldrich, USA). The RNA concentration and purity were determined spectrophotometrically (Maestrogen, Hsinchu, Taiwan), and only high-purity samples (A260/280 ≥ 1.9) were exploited for downstream analysis. Reverse transcription was performed by way of a High-Capacity cDNA Reverse Transcription Kit (Applied Biosystems, USA) in a Mastercycler Gradient thermal cycler (Eppendorf, Hamburg, Germany) using the following program: 25 °C for 10 min, 50 °C for 50 min and 80 °C for 5 min. Each reaction was performed in triplicate.

#### Quantitative real-time PCR

Relative mRNA expression was quantified by way of a PowerUp SYBR Green Master Mix (Thermo Fisher Scientific, USA) on a 7500 Real-Time PCR System (Applied Biosystems, USA). Gene-specific primers (Genomed, Warsaw, Poland) were designed based on NCBI sequences via Primer3 (v.0.4.0); the sequences are listed in Table 1. The ubiquitin-conjugating enzyme gene *UBC13* was used as the sole reference gene. Its expression stability was not formally evaluated either between untreated and THY-treated samples within each growth state or across planktonic and biofilm-forming conditions in the present isolate set. ΔCt was calculated for each target gene. For the planktonic cultures, the corresponding untreated planktonic sample was used as the calibrator, whereas for the biofilm cultures, the corresponding untreated biofilm sample served as the calibrator. Relative expression was calculated as RQ = 2^−ΔΔCt. The technical quality control of the qPCR assays was based on amplification curves, melting temperatures and Ct values. The results were expressed as mean RQ ± SD.

**Table 1.** Primer sequences and product lengths for analysis of *C. albicans* gene expression using RT-qPCR.

Gene name	Primer sequence	Product length (bp)
*ALS3*	Forward: 5′-GTGATGCTGGATCTAACGGTATTG-3′	112
	Reverse: 5′-GTCTTAGTTTTGTCGCGGTTAGG-3′	
*CYS3*	Forward: 5′-GTGGTATCGAGTCGTTGATCGA-3′	80
	Reverse: 5′-ACCATTGGCTTCTCTTTCTTCCT-3′	
*HWP1*	Forward: 5′-TCAGTTCCACTCATGCAACCA-3′	99
	Reverse: 5′-AGCACCGAAAGTCAATCTCATGT-3′	
*NRG1*	Forward: 5′-CACCTCACTTGCAACCCC-3′	198
	Reverse: 5′-GCCCTGGAGATGGTCTGA-3′	
*SAM2*	Forward: 5′-GGTTCCTTGCCATGGTTGAG-3′	97
	Reverse: 5′-TTGTGTCGACTCTTTTTGGGATAA-3′	
*TUP1*	Forward: 5′-GCTTCAGGTAACCCATTGTTGAT-3′	88
	Reverse: 5′-CTTCGGTTCCCTTTGAGTTTAGG-3′	
*GRF10*	Forward: 5′-ACACCTACTCCGCCAACATC-3′	150
	Reverse: 5′-ACGTTCAGGACTTGGGTTTG-3′	
*BCR1*	Forward: 5′-CTTCAGCAGCTTCATTAACACCTA-3′	101
	Reverse: 5′-TCTTGGATCAGGTGTACTTTTCAA-3′	
*UBC13*	Forward: 5′-GAAAGATAATTGGTCACCCGCAT-3′	82
	Reverse: 5′-ATCTGGGTTTGGAGCCCCT-3′	

### Data analysis

All the samples were analyzed in triplicate (technical replicates within a single isolate), and representative data: mode (for antifungal effect, antibiofilm effect, mechanism of action and influence on filamentation) or mean ± standard deviation (SD) (for antibiofilm effect) are shown. Regarding the antibiofilm activity of THY, the percentage of biofilm inhibition and reduction was determined as mean ± SD for both reference *C. albicans* strains. In turn, the inhibition of biofilm formation by ≥50 and ≥90%, or its reduction by ≥50 and ≥90% in the presence of THY, was presented as a mode value for all clinical *C. albicans* isolates. Statistical analysis was performed via Statistica 10 software (StatSoft Statistica, Poland). For the analysis of relationship between values of MIC, MFC, and concentration values of ≥50 or ≥90% inhibition, as well as THY-induced reduction of total biofilm biomass, a nonparametric Spearman rank correlation test was applied. Statistical significance was set at *p* ≤ 0.05.

RT-qPCR data was analyzed using ΔCt values. For each isolate, technical triplicates were averaged prior to assessment. Differences between untreated and THY-treated samples within the same growth condition (planktonic or biofilm) were rated by way of the Wilcoxon matched-pairs signed-rank test. Because eight genes were evaluated within each growth condition, *p*-values were adjusted for multiple comparisons using the Benjamini‒Hochberg false-discovery-rate procedure separately for the biofilm-associated and planktonic analyses. Both unadjusted *p*-values and adjusted *q*-values are reported. The RT-qPCR analysis was considered as exploratory, and *q* < 0.05 was established as the threshold for statistical significance. Descriptive statistics are presented as median ΔCt, mean ΔCt ± standard deviation (SD), and corresponding *p*-values. Descriptive statistics, raw *p*-values, and multiple-testing-adjusted *q*-values are reported separately for biofilm-associated and planktonic cells in Supplementary Tables S2 and S3, respectively (Table 2).

**Table 2.** Characteristics and role of the studied genes in biofilm formation.

Gene	Characteristics and role of the studied genes in biofilm formation	Literature data
*ALS3*	*ALS3* (Agglutinin-Like Sequence 3) gene encodes the Als3 protein; plays a key role in adhesion, binding of fungal cells to host epithelial cells, and extracellular matrix proteins, enables the interaction of the fungus with host tissues, invasion, penetration of *C. albicans* into cells and development of infection	[35–39]
*HWP1*	*HWP1* (hyphal wall protein 1) gene encodes major hyphal wall protein Hwp1; holds an important role as adhesin *C. albicans* in attachment to host epithelial cells; is required for normal hyphal development, cell wall assembly and intracellular signaling	[35,38–42]
*TUP1*	*TUP1* gene encodes the transcriptional repressor Tup1, which enacts two key roles in the cell: regulation of phase switching, and inhibition of filamentous growth	[41,43–45]
*NRG1*	*NRG1* gene encodes the transcriptional regulator Nrg1, a key regulator of *C. albicans* filamentation and hyphal growth, as well as in the expression of hyphal-specific genes, virulence genes and chlamydospore formation; influences multiple different steps during the *C. albicans* biofilm developmental cycle, yeast–hypha morphogenesis and stress response	[43,44,46,47]
*GRF10*	*GRF10* gene encodes a homeodomain containing transcription factor Grf10, which is an important regulator of both the morphogenesis and metabolism of *C. albicans*, is necessary for the yeast-to-hypha morphological switching, filamentation, chlamydospore and hyphae formation	[48–50]
*BCR1*	*BCR1* gene encodes the transcription factor Bcr1, acts as a master regulator of biofilm formation; activates cell-surface protein and adhesin genes, including several induced during hyphal development, especially Als3 and Hwp1	[39,51–53]
*SAM2*	*SAM2* (S-adenosylmethionine) gene encodes enzyme S-adenosylmethionine (SAM) synthetase, which is the major methyl donor for methylation events of proteins, biotin, RNAs and lipids; also plays a role in the amine production used in detoxification of reactive oxygen species (ROS) and in the maintenance of the redox status in cells	[54,55]
*CYS3*	*CYS3* (Cystathionine γ-Lyase) gene encodes cystathionine γ-lyase (CYS3), an enzyme in the sulfur amino acid pathway that converts cystathionine to cysteine. It generates hydrogen sulfide (H_2_S), which helps *C. albicans* grow, transforms harmless yeast cells into invasive hyphae, protects fungal proteins from reactive oxygen species (oxidative stress response) and also affects the structure of the cell wall and the formation of biofilm	[56]

## Results

### Antifungal activity of THY

The obtained data indicated the potential antifungal effect of THY towards yeasts from *C. albicans* belonging to 2 reference strains: *C. albicans* ATCC 10231 and *C. albicans* ATCC 2091, as well as 30 clinical isolates drawn from hospitalized patients with hematological malignancies.

The evaluation of the anticandidal activity of THY showed that the MIC against both reference strains was in the range of 125–250 μg/mL, while the values of MFC ​​were identical or 2–4 times higher and amounted to 125–500 μg/mL. According to O'Donnell et al. [23], antimicrobial activity of substances at MIC value in the range of 126–500 μg/mL are defined as moderate, and good at a MIC range of 26–125 μg/mL. Thus, THY had moderate or good antifungal activity. It should be also added that a beneficial fungicidal effect of MFC/MIC = 1–4 was noticeable against these strains.

In the next stage of our research, the activity of THY was confirmed towards clinical isolates of *C. albicans* derived from the oral cavity of hemato-oncological patients (such individuals are particularly vulnerable to candidiasis). The results presented in Figure 1 demonstrate that the anticandidal activity of THY was evident at MIC = 62.5–500 μg/mL. The MFC values were slightly higher, in the range of 125–1,000 μg/mL. According to the distribution of MIC values of THY among these strains, *C. albicans* was most inhibited at a concentration of 125 μg/mL (12 (40%) isolates). Sensitivity to this compound at MIC of 250 μg/mL and 500 μg/mL were found to be at a frequency of 10 (33.3%) and 2 (6.7%) isolates, respectively. Particularly interesting was the susceptibility to THY at a concentration of 62.5 μg/mL, which was observed in 6 (20%) strains.

**Figure 1.**
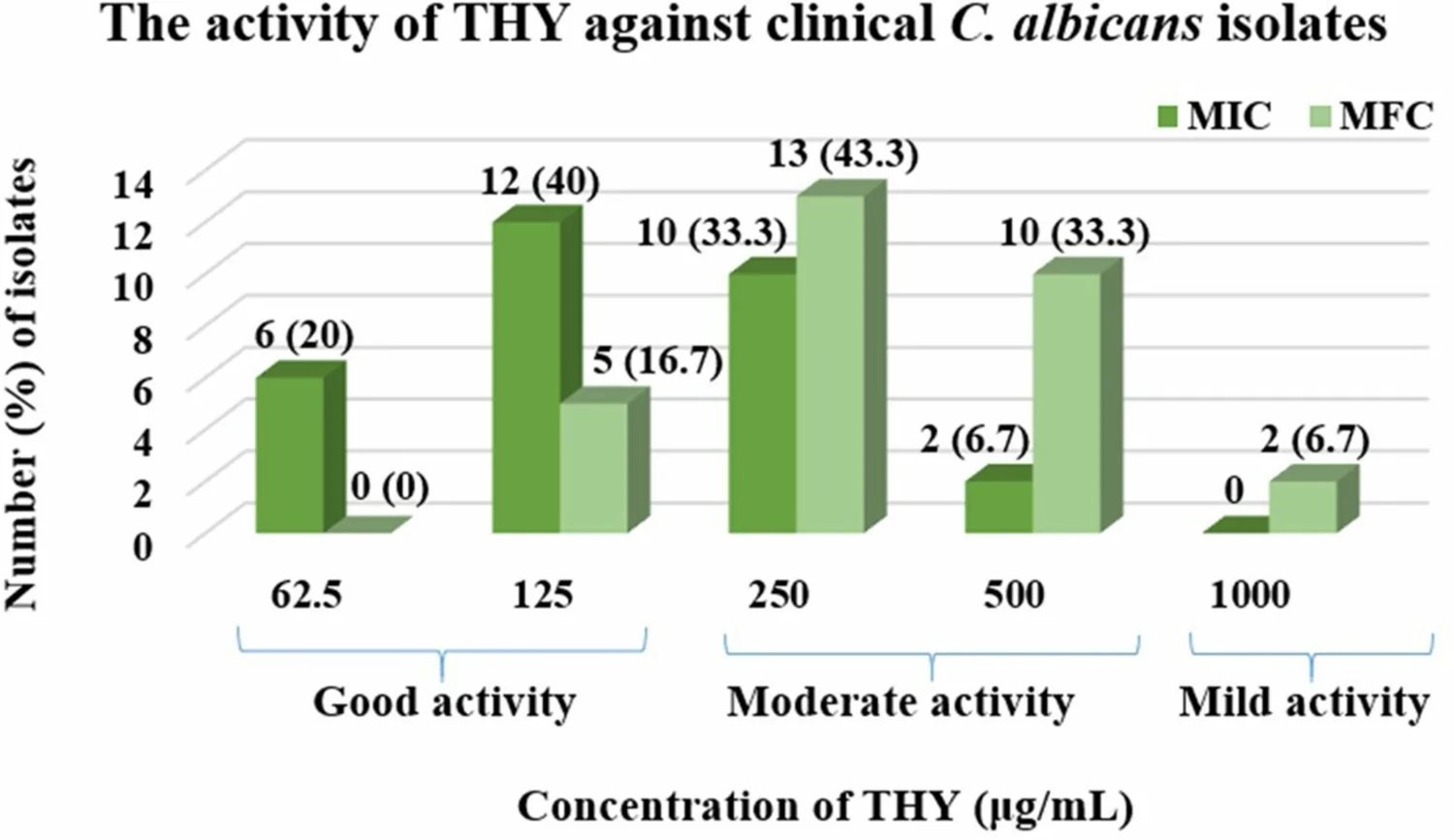
Distribution of MIC and MFC values [μg/mL] and degree of antifungal activity of THY towards 30 clinical isolates of *C. albicans* from hospitalized patients with hematological malignancies.

In the case of MFC, the values were, as before, the same or 2–4 × higher than MIC. The minimum THY concentrations that killed most of the tested isolates (13 (43.3%) and 10 (33.3%) strains) were 250 μg/mL and 500 μg/mL, respectively. In turn, MFC at 125 μg/mL was fungicidal for 5 (16.7%) isolates, while 1,000 μg/mL was effective against 2 (6.7%) strains. Taking into account the degree of antifungal activity, THY revealed good bioactivity towards 18 (60%) (most strains) and moderate activity against 12 (40%) clinical *C. albicans* strains (Figure 1).

We also analyzed THY MIC_50_ and MIC_90_ values. These are the minimum concentrations inhibiting the growth of 50 or 90% of all *C. albicans* strains. The derived values ​​were: MIC_50_ = 125 μg/mL and MIC_90_ = 250 μg/mL. The MFC_50_ and MFC_90_ values, i.e. the lowest concentrations killing 50 or 90% of tested isolates, were also assessed. Herein, MFC_50_ = 250 μg/mL and MFC_90_ = 500 μg/mL. In addition, the MFC/MIC ratio was determined based on the MIC and MFC values. The obtained data showed that THY has fungicidal activity (MFC/MIC ≤ 4) towards all clinical *C. albicans* strains drawn from the oral cavity of hemato-oncological patients.

### Influence of THY on *C. albicans* biofilm formation and mature biofilm

Our results showed that reference strains of *C. albicans* had moderate ability to produce biofilm. In the case of 30 clinical isolates, the ability to form this structure varied. Of these, 10 (33.3%) strains exhibited strong biofilm production, and the remaining 16 (53.3%) and 4 (13.3%) isolates were moderate and weak biofilm producers, respectively.

The effect of THY (in two-fold increasing concentrations ranging from 15.62 to 8,000 μg/mL) on the inhibition and reduction of total biofilm biomass was analyzed using crystal violet staining. The quantification of total biomass included both living cells, dead cells and matrix material. As shown in Figures 2A–D, 3A and B, THY induced inhibition and reduction of biofilm in a concentration-dependent manner. For both reference strains, an inhibitory and reducing effect of THY on biofilm biomass was observed from concentrations of 15.62 μg/mL (3.67–8%) and 31.25 μg/mL (12.33–19.67%), respectively. The concentrations required to inhibit ≥50 and ≥90% biofilm formation of these strains were higher: from 250 μg/mL to 1,000 μg/mL (Figure 2A and C). In turn, ≥50% reduction of the biofilm of both strains occurred at a concentration of THY twice as high (from 500 μg/mL) as its inhibition, while the THY concentrations that reduced ≥90% of the biofilm were 2,000 μg/mL for *C. albicans* ATCC 10231 and 4,000 μg/mL for *C. albicans* ATCC 2091 (Figure 2B and D). Experiments comparing the total biofilm biomass of the two *C. albicans* strains conducted in these studies showed that 2- or 4-fold higher concentrations of THY were required for significant removal of mature biofilm than for its inhibition (Figure 2A–D).

**Figure 2.**
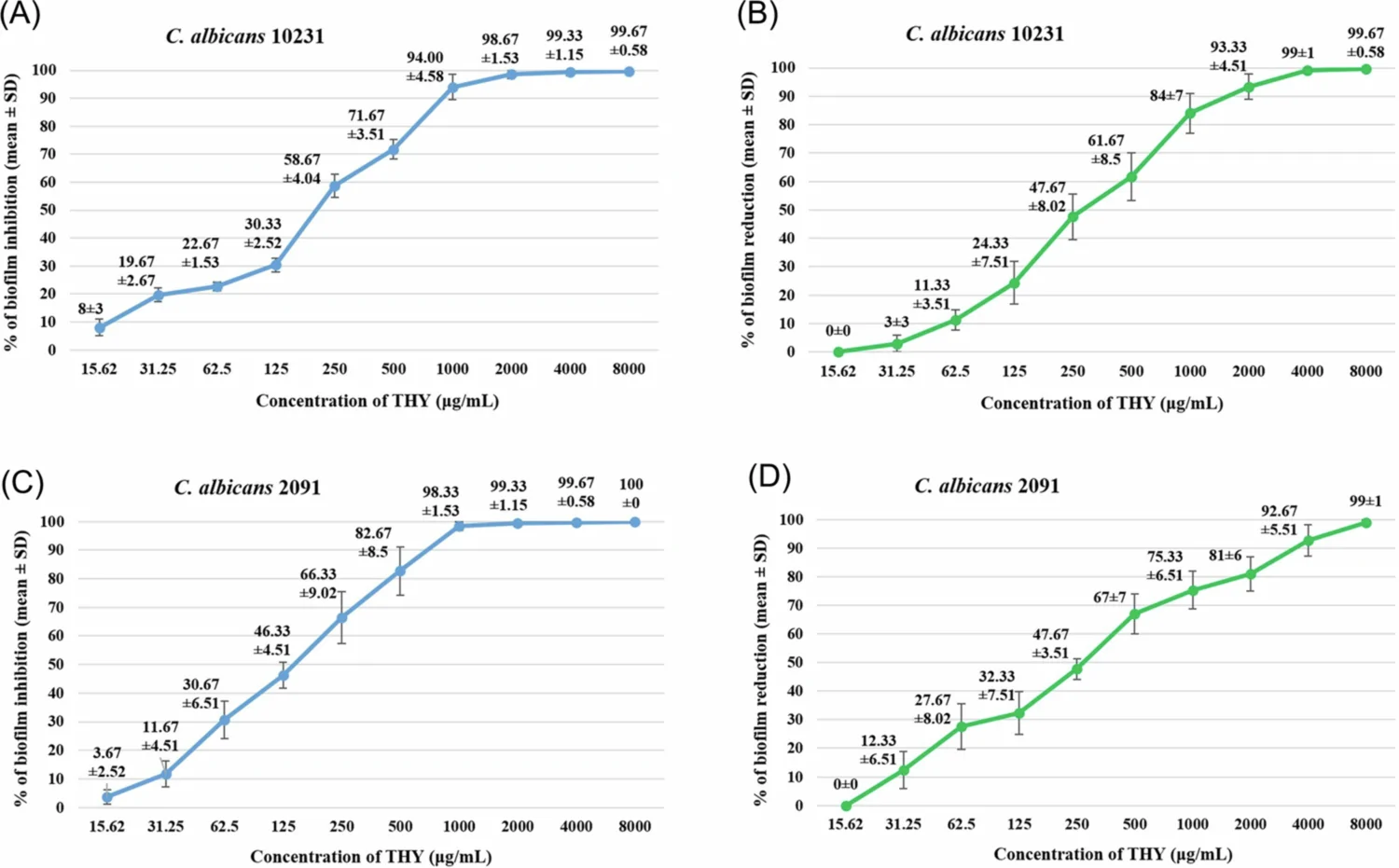
Effects of different concentrations of THY on biofilm formation and mature biofilm of *C. albicans* ATCC 10231 (A, C) and *C. albicans* ATCC 2091 (B, D).

**Figure 3.**
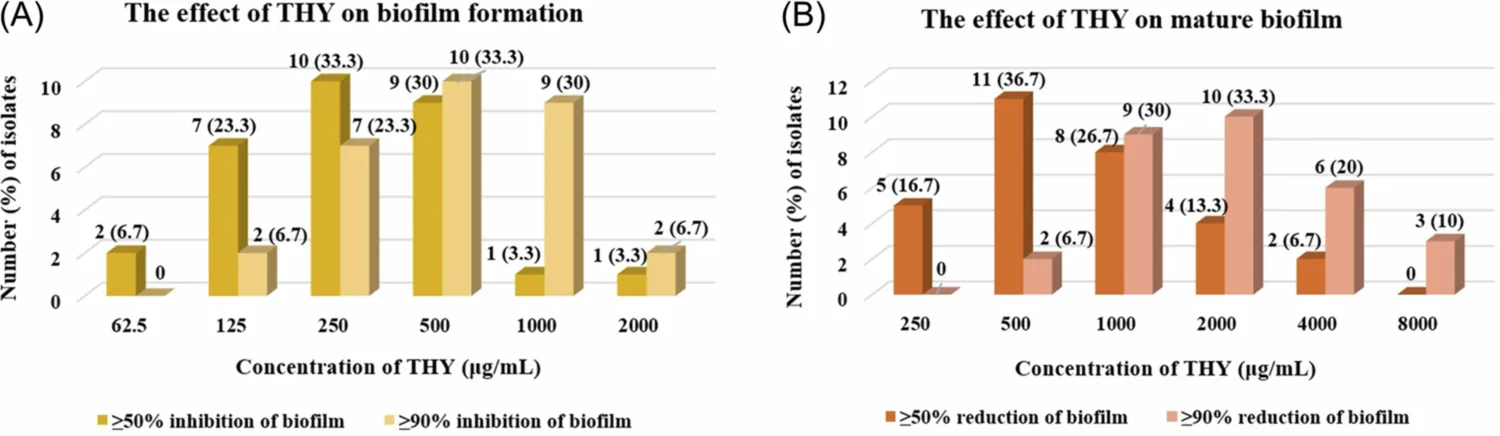
Effects of different concentrations of THY on biofilm formation (A) and mature biofilm, (B) of clinical isolates of *C. albicans*.

These investigations were continued with clinical strains of *C. albicans*. The concentrations of THY required to inhibit ≥50% biofilm formation of isolates were in the range of 62.5–2,000 μg/mL, and for ≥90% inhibition of biofilm growth, the concentrations were 125–2,000 μg/mL. Biofilm inhibition of ≥50 and ≥90% occurred most frequently in 10 (33.3%) isolates tested at concentrations of 250 μg/mL and 500 μg/mL, respectively (Figure 3A). In turn, the values of ≥50% biofilm reduction against the same clinical strains ranged from 250 to 4,000 μg/mL, while ≥90% biofilm reduction was from 500 to 8,000 μg/mL. Moreover, the biofilm of 11 (36.7%) isolates was reduced at ≥50% at a concentration of 500 μg/mL, while in 10 (33.3%) isolates, the reduction of biofilm at ≥90% was at a concentration of 2,000 μg/mL. Studies including the effect of THY on biofilm in 30 *C. albicans* strains also showed that higher THY concentrations (4- or 32-fold) were necessary to significantly remove existing biofilm cells than to inhibit it (Figure 3B). These results demonstrate that the biofilm structure provides a several-fold increase in the insensitivity of *C. albicans* to THY.

The obtained results were assessed using the Spearman's rank correlation test, which is a nonparametric measure of the strength and direction of the relationship between two variables. In our work, Spearman's rank correlation coefficient (R, rho) was applied to examine the connection between values of MIC or MFC and concentration values of ≥50 or ≥90% inhibition, as well as THY-induced reduction in total biofilm biomass. Statistical significance was set at *p* < 0.05. Using this test, a strong positive correlation was demonstrated between MIC and concentration induced ≥50% inhibition of biofilm (R = 0.82, *p* = 0.01), MFC and values of ≥50% inhibition of biofilm (R = 0.85, *p* = 0.01), and between MFC and concentration ≥90% inhibition of biofilm (R = 0.79, *p* = 0.02). These values ​​were also statistically significant (*p* < 0.05). In turn, no correlation was found between MIC or MFC values ​​and concentrations ≥50 or ≥90% reduction of total biofilm biomass. This means that an increase in the MIC or MFC value was associated with an increase in the ≥50 or ≥90% of inhibition of total biofilm biomass values, respectively.

### Mechanism of antifungal action of THY

Subsequent results revealed a potential mechanism of action for THY. Accordingly, sorbitol testing was applied to determine whether THY affects the structure of *C. albicans* cell wall. In turn, the ergosterol test was aimed at determining the effect of THY on the yeast cell membrane (Figure 4A).

**Figure 4.**
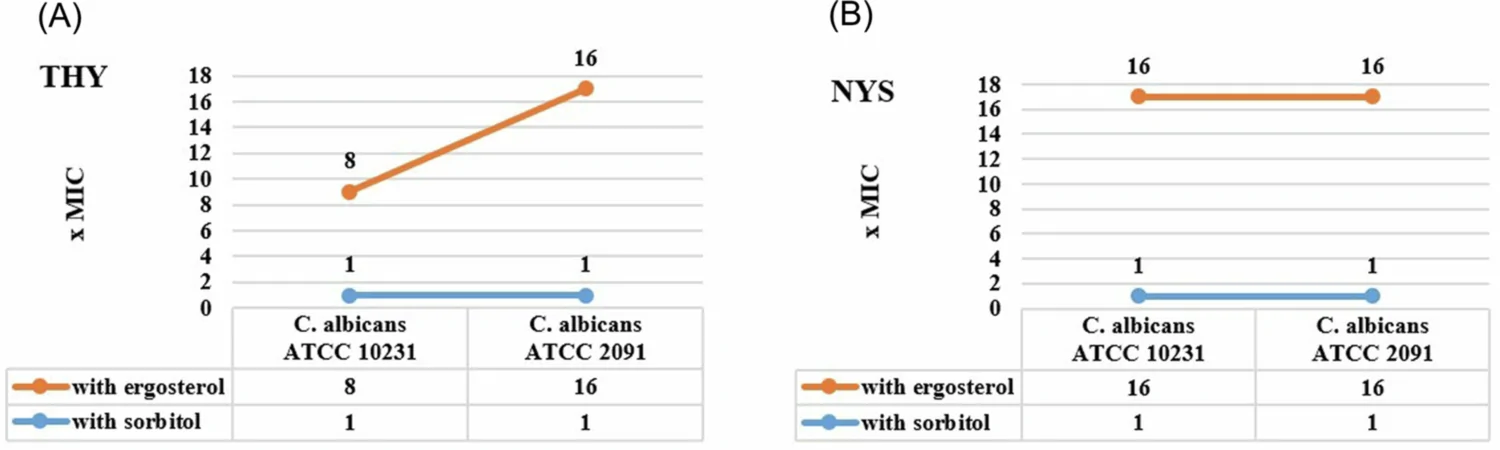
The increase in the MIC values (×MIC) of THY (A) and nystatin (NYS) (as control), (B) in the presence of ergosterol (400 μg/mL) and sorbitol (0.8 M) against both reference *C. albicans* strains.

Sorbitol has important osmoprotective properties. It is crucial for the growth of fungi and serves to stabilize fungal protoplasts by protecting their cell walls against osmotic ch.anges. THY MIC assessment was performed simultaneously on separate plates with and without sorbitol. In the assessment, in the case of a positive test, the MIC value of the cell wall damaging compound will be significantly higher in the presence of osmotic support. Our results, shown in Figure 4A, indicated that the MIC did not change in the presence of sorbitol even after 7 days of incubation in any of the studied strains. These findings suggest that the antifungal activity of THY is probably not related to the inhibition of the mechanisms responsible for cell wall synthesis. The MIC of NYS, which acts on the fungal cell membrane, was also unchanged in media with sorbitol (Figure 4B).

The study continued with another assay to determine whether THY affects ergosterol in the *C. albicans* cell membrane. This sterol is an important target for several classes of antimycotics used in the treatment of candidiasis. The applied test detects whether THY can bind to ergosterol in the cell membrane of fungi, and the method is based on the delivery of exogenous ergosterol to the medium. Herein, if the antifungal compound has an affinity for ergosterol, it quickly forms a complex with it. In this way, it prevents the formation of a complex with membrane ergosterol. As a result, the MIC value increases. The obtained data (Figure 4A) indicated higher MIC values ​​of THY in the medium supplemented with exogenous ergosterol compared to the medium without ergosterol. These values ​​increased up to 8–16 times in the presence of the sterol. In the case of NYS (positive control) (Figure 4B), the mechanism of action of which is based on interaction with ergosterol, a 16-fold higher MIC value was confirmed. Our results, which were based on reference strains, suggest that THY may likely have an effect on the cell membrane. Disturbed synthesis of this sterol increases ion permeability and leads to fungal cell death.

### Influence of THY on *C. albicans* filamentation process

The filamentation test showed that THY, depending on its concentration, is able to inhibit the filamentation process of *C. albicans* (Figure 5A and B). In contrast, in the control (without THY), *C. albicans* cells showed the ability to generate intense filamentation with numerous blastopores and chlamydospores. It should also be emphasized that the concentration of 1/4 MIC (31.25 μg/mL), THY did not completely inhibit the filamentation process but only weakened it. A much beneficial effect can be seen for the concentrations of 1/2 MIC (62.5 μg/mL), where the filamentation process was almost completely inhibited. higher activity with inhibition effect of germination was observed at MIC (125 μg/mL).

**Figure 5.**
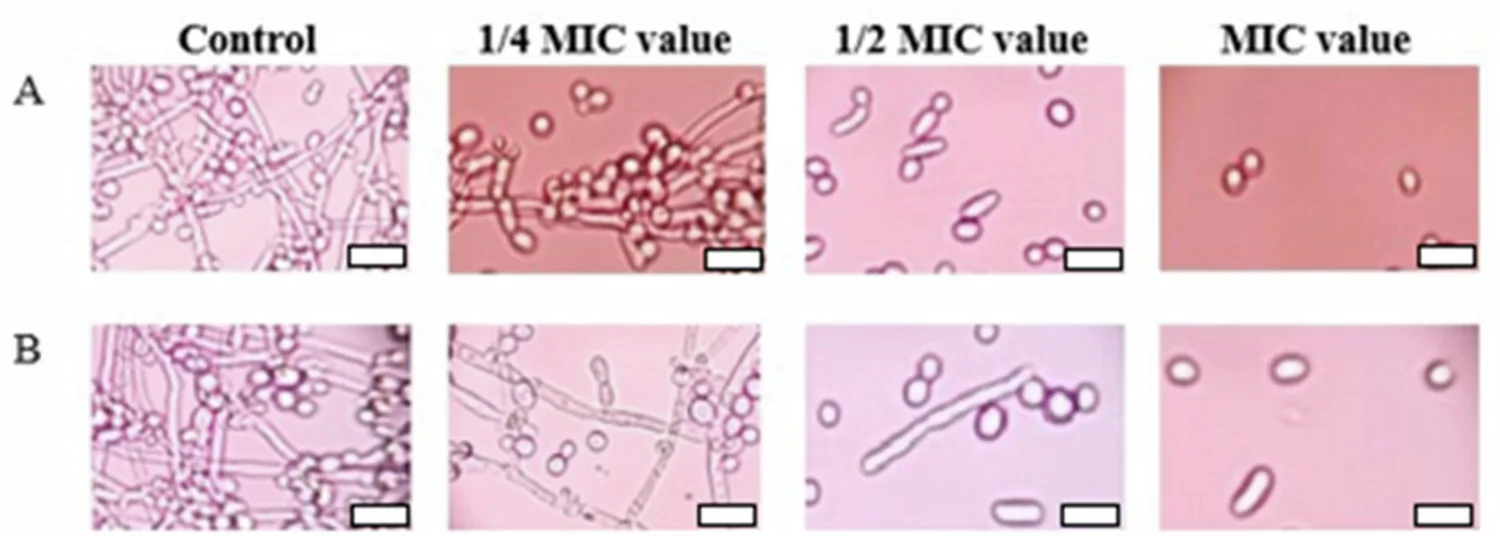
The influence of THY (in various concentration: 1/4 MIC, 1/2 MIC, MIC) on *C. albicans* ATCC 10231 (A) and *C. albicans* ATCC 2091, (B) filamentation process. Scale bar = 10 μm.

### Effect of THY on the expression of genes responsible for *C. albicans* adhesion and biofilm formation

In this experiment, from 30 tested *C. albicans* strains isolated from immunosuppressed patients, 10 (33.3%) isolates belonging to strong biofilm producers (4 × ODc < OD) were selected and used for further studies. For these isolates, the MIC and MFC values ​​of THY ranged from 125 to 500 μg/mL and 250 to 1,000 μg/mL, respectively. In turn, the values of ≥50% biofilm inhibition were in the range of 250 to 2,000 μg/mL, while ≥50% biofilm reduction in the range of 500 to 4,000 μg/mL (Supplementary Table S1).

In all strains, the presence of 8 genes responsible for adhesion and biofilm formation was previously confirmed *in vitro*: *ALS3, CYS3, HWP1, NRG1, SAM2, TUP1, GRF10* and *BCR1*. These genes are associated, among others, with adhesion, biofilm formation, hyphal growth and amino acid metabolism. As we had noticed that THY had some effect on biofilm inhibition and formation, we, therefore, set out to assess its influence on the expression of these genes in *C. albicans*. The level of mRNA expression of genes involved in biofilm development was determined after THY treatment of *C. albicans* strains. The results of the effects of THY on planktonic and biofilm cells were analyzed separately (Figure 6).

**Figure 6.**
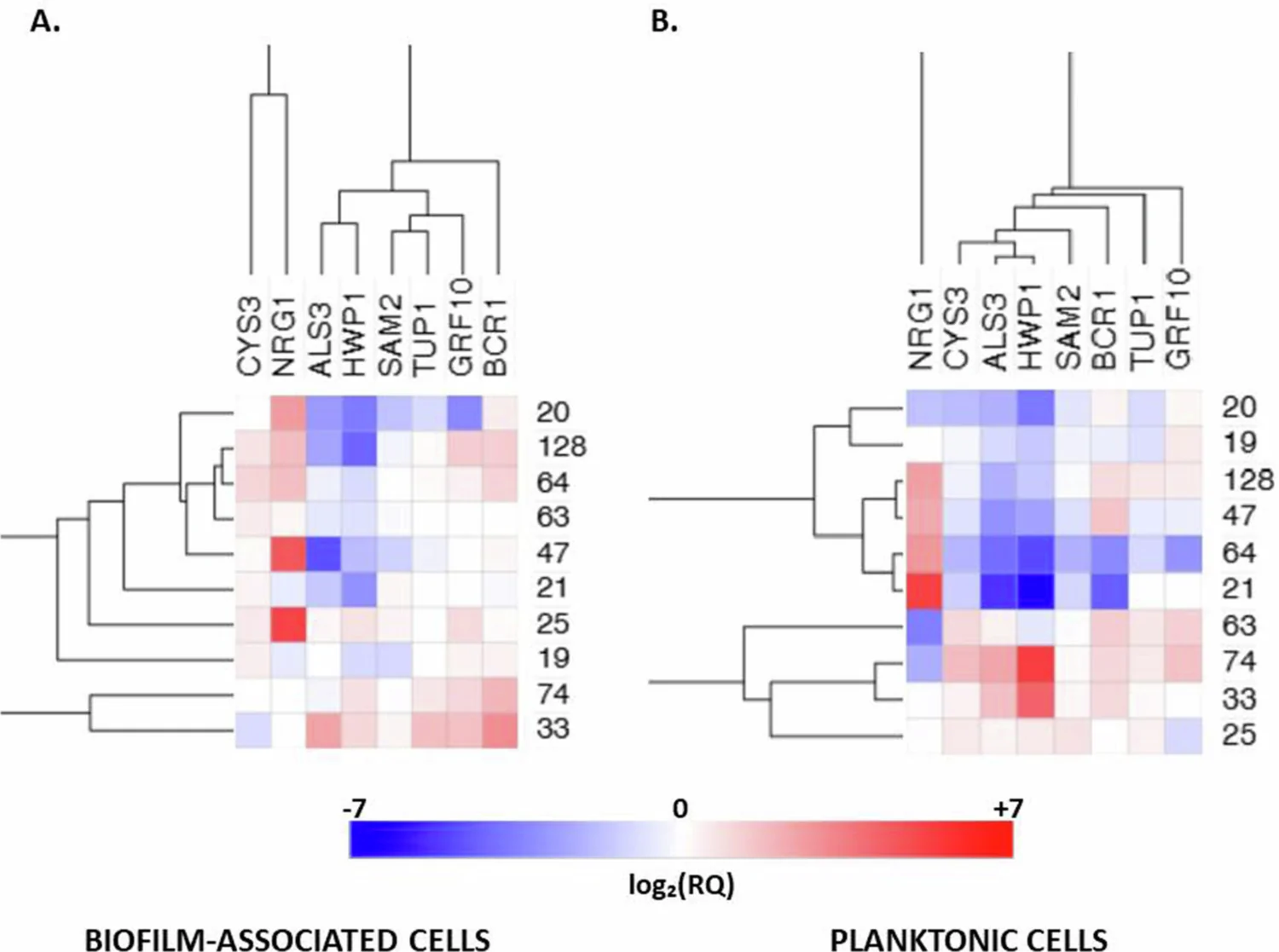
Hierarchical clustering of thymol-induced gene expression changes in ten strong biofilm-producing *Candida albicans* clinical isolates under (A) biofilm-forming and (B) planktonic conditions. The relative expression for each isolate and gene was calculated as RQ = 2^−ΔΔCt, using the untreated sample from the corresponding growth condition as the calibrator, and is presented as log_2_(RQ). A fixed color scale from −7 to +7 log_2_(RQ) was applied to both panels: blue indicates negative values, white indicates no change (log_2_[RQ] = 0), and red indicates positive values. Rows correspond to clinical isolates, and columns to genes. Hierarchical clustering was conducted using one-minus Pearson correlation and average linkage. For visualization, each row and column dendrogram was cut immediately below its final merge, corresponding to the highest-level bifurcation and resulting in two major dendrogram-defined groups (k = 2). The heatmaps represent treatment-induced changes relative to condition-matched untreated controls and do not indicate basal expression levels. The dendrogram-defined groups were not inferentially validated as distinct biological response strategies.

To further evaluate the observed transcriptional patterns, statistical analysis was performed using ΔCt values derived from individual isolates. The analysis of paired ΔCt values is summarized in Supplementary Table S2 for biofilm-associated cells and Supplementary Table S3 for planktonic cells. In biofilm-associated cells, *BCR1* yielded the smallest unadjusted *p*-value (*p* = 0.010), but this result did not remain statistically significant after Benjamini‒Hochberg adjustment (*q* = 0.080). In addition, *ALS3* and *NRG1* (both *p* = 0.065, *q* = 0.141) and *HWP1* (*p* = 0.084, *q* = 0.141) displayed non-significant directional trends, and no gene showed a statistically significant change in planktonic cells (all unadjusted *p* ≥ 0.193; all *q* = 0.770). Because treatment-by-growth-state differences were not tested directly, the separate within-condition analyses were not used to infer a differential THY response between growth states. Beyond the aforementioned, no statistically significant within-condition changes were detected in planktonic cells. Here too, because treatment-by-growth-state differences were not tested directly, separate within-condition analyses were not employed to infer a differential THY response between planktonic and biofilm-associated cells.

A descriptive comparison based on the condition-specific median ΔCt values is found in Supplementary Table S2. Here, it can be seen that *ALS3* showed the largest median-derived decrease (ΔΔCt = +2.10; RQ = 0.23, approximately 4.3-fold lower), whereas *NRG1* revealed the largest median-derived increase (ΔΔCt = −2.25; RQ = 4.76). Moreover, *BCR1* demonstrated a smaller median-derived increase (ΔΔCt = −1.51; RQ = 2.84) and the smallest unadjusted *p*-value; however, this association did not remain statistically significant after adjustment (*q* = 0.080). No other gene approached the adjusted significance threshold, and no gene indicated a significant change in planktonic cells. Thus, the magnitude of the descriptive fold change did not directly correspond to the strength of the paired statistical evidence.

Hierarchical clustering of log_2_(RQ) values, which represent THY-induced expression changes relative to condition-matched untreated controls, revealed substantial isolate-to-isolate heterogeneity (Figure 6AB). Under biofilm-forming conditions (Figure 6A), the dendrogram identified two major isolate clusters, with additional variation within the larger cluster. Under planktonic conditions (Figure 6B), a different grouping of isolates was observed. Because the displayed values are normalized treatment effects, the heatmaps do not provide information on basal gene expression. The cluster structure is descriptive and should not be interpreted as evidence of statistically established transcriptional strategies. Hierarchical clustering analysis of log2-transformed relative expression values demonstrated considerable heterogeneity in the transcriptional response of *C. albicans* clinical isolates to THY exposure (Figure 6AB). Under biofilm-forming conditions (Figure 6A), the isolates were grouped based on similarities in their gene expression profiles, suggesting two predominant response patterns. The first pattern, represented by isolates 20, 128, 64, 63 and 47, was characterized by decreased expression of *ALS3* and *HWP1* accompanied by increased expression of *NRG1* and *BCR1*. In contrast, isolates 74 and 33 displayed a distinct expression profile, while isolates 21, 25 and 19 occupied an intermediate position within the dendrogram. Gene-based clustering revealed that *HWP1, SAM2, TUP1* and *GRF10* exhibited relatively similar expression patterns across the biofilm isolates analyzed, whereas *CYS3* showed the most distinct transcriptional profile. A comparable degree of heterogeneity was observed among the planktonic cells (Figure 6B). Similar to the biofilm-associated population, *ALS3* and *HWP1* frequently exhibited reduced expression following THY exposure, whereas *BCR1* and *GRF10* tended to display increased expression in a subset of isolates. However, substantial isolate-to-isolate variability was evident under both growth conditions.

Overall, the clustering analysis indicated that THY-induced transcriptional responses were strain-dependent and that individual clinical isolates differed considerably in the magnitude and direction of gene regulation.

## Discussion

### Effect of THY on planktonic and biofilm-associated cells of *C. albicans*

The frequent occurrence of opportunistic fungal infections caused by *C. albicans*, especially in predisposed patients, indicates the need to search for new therapeutic solutions. This is very important because these fungi are characterized by high morphological plasticity and the ability to express genes involved in biofilm formation and acquiring resistance to antimycotics [57]. Therefore, our studies evaluated the antifungal activity of THY against *C. albicans.*

In terms of the anticandidal effect of THY, large contrasting discrepancies in MIC values were found, depending on the authors cited. This natural compound exhibited excellent effect in some studies (even from 1.56 μg/mL) [58], while in others, the activity was very poor (up to 80,000 μg/mL) [9,59]. Such variations may be related, for example, to differences in the purity and in the quality of the tested compound [35]. Additionally, differences in the methods used to determine MIC values, i.e. test protocol, type of culture medium, pH of the medium, inoculum size, density of the fungal suspension, incubation conditions (temperature or time) and interpretation criteria, may influence the observed MIC values ​​and lead to diversity in the results. The antifungal activity of THY may also be dependent on the type of strain used in the study, as clinical isolates of *Candida* exhibit varying susceptibility to drugs, antiseptics or chemicals [9,35,59]. Therefore, when interpreting MIC values, the context of a specific study and other results contained in the broader literature should be taken into account [35].

The obtained data shows that THY has an antifungal effect against both reference *C. albicans* strains and those isolated from the oral cavity of patients with hematological malignancies being of high risk of candidiasis development. The data indicates that the tested strains are susceptible to THY at MIC values ​​ranging from 62.5 to 500 μg/mL. This good and moderate bioactivity with MFC/MIC ≤ 4 and fungicidal effect of THY was confirmed against all studied *C. albicans* isolates. Many results of other authors showed the same or similar activity towards this species. According to most [35,57,60–62], anticandidal activity of THY was also good or moderate at values of MIC = 32–150 μg/mL. Based on later data, the growth inhibition of *C. albicans* occurred at similar THY concentrations but in a wider ranges of 32–256 μg/mL [40,43,62–65] and 50–400 μg/mL [66]. Further studies showed that this phytoconstituent was active at slightly higher MIC at 500 μg/mL [67], indicating its moderate to mild activity. MFC values ​​of THY towards *C. albicans* were also similar to ours and within the range of 62.5–87.5 μg/mL [57] or 250–256 μg/mL [63,64]. In turn, other data showed MFC = 400–2,000 μg/mL [66,67] for these yeasts.

Our studies were continued to determine the antibiofilm effect of THY. This activity can be assessed using various methods. These include the tetrazolium salt (MTT) and resazurin tests, which allow for the estimation of cell viability by measuring the metabolic activity of living cells in a biofilm. Another method is that of quantification of the total biofilm biomass (live cells, dead cells and matrix material) using CV staining. The method we employed showed that the concentrations of THY required to inhibit ≥50 or ≥90% of this structure are 62.5–2,000 μg/mL, depending on the strain. In turn, the values ​​necessary to reduce ≥50 and ≥90% of the total biomass are much higher (4 or 32-folds) and range from 250 to 8,000 μg/mL, respectively. Given its ability to grow in biofilms, *C. albicans* may tolerate higher concentrations of THY.

The results of other authors assessing the antibiofilm activity of THY were based on different methods. Miranda-Cadena et al. [40] showed that THY may be a potential alternative for eradicating yeasts biofilms. In their work, THY reduced at 24 h more than 80–85% of *C. albicans* biofilms metabolic activity of mature biofilms. These data are in line with those described by other researchers [67,68], who observed a reduction of >80% in the metabolic activity of *C. albicans* biofilm after 24 h of incubation with THY. Similarly, Dalleau et al. [69] indicated that mature biofilms of *C. albicans* isolates were also susceptible to THY. Further data [63] showed that in a study using CV staining, the 24-h biofilm biomass was 63 and 68% after treatment with THY at concentrations of 250 μg/mL and 125 μg/mL, respectively. Abu Shaban and Darmani [35], for example, observed an antibiofilm effect of THY that resulted in 55% inhibition of this structure at sub-inhibitory concentration. Other authors confirmed the differential inhibitory effects of THY against *C. albicans* biofilm at concentrations of 32 μg/mL [43] and 2 mg/mL [67]. Moreover, data presented by Jafri and Ahmad [58] revealed that at a THY concentration of 3.12 mg/mL, the rate of biofilm formation by *C. albicans* was 16.93%. In turn, the concentration of THY eradicating 80% of pre-formed biofilms varied from 3.12 mg/mL to 25 mg/mL [58]. Other results [70] indicated that THY's effect on cell adhesion was dose-dependent. Herein, at a concentration of 1 × MIC, the biomass adhesion was reduced by 61.3% and at 2 × MIC by 81.7%. Finally, studies conducted by Sharifzadeh et al. [57] showed a reduction in *C. albicans* biofilm mass by up to 82% caused by THY. The data mentioned above confirmed the antibiofilm activity of THY.

Comparing the inhibitory and fungicidal concentrations of THY against planktonic and biofilm-associated cells revealed that these values were different. For biofilm-associated cells, significantly higher THY concentrations are required to inhibit or reduce them. Therefore, the use of THY for preventive inhibition of biofilm formation may be a promising approach [70]. It should be noted that in our work, Spearman's test revealed a strong positive correlation (R = 0.79–0.85) between MIC, MFC and concentration values ≥50 or ≥90% inhibition of total biofilm biomass. This means that an increase in the MIC or MFC value is associated with an increase in biofilm inhibitory values. Additionally, the obtained data were statistically significant (*p*< 0.05). These results indicate that higher THY concentrations are required to reduce biofilm biomass than to inhibit planktonic growth. However, because biofilm susceptibility was assessed using CV staining, which measures total biomass rather than viability, the observed differences should be interpreted as a reduced susceptibility of the biofilm structure rather than direct evidence of increased survival of biofilm-associated cells. Data from the literature [7,57,58] reveals that biofilms exhibit increased resistance to antimycotics as well as resistance to host defense, which leads to the failure of conventional antifungal therapy. In the biofilm mode of growth, yeast cells display characteristic traits different to the planktonic state. This complicates its eradication, enhances mortality during infection, prolongs hospital stays and increases the cost of antifungal therapy [9].

### Mechanism of action of THY on *C. albicans* cells

Some reports have shown that selected compounds of essential oils can affect the cellular activities of yeast, leading to their death [11,71]. However, only preliminary data explaining the mechanisms of this action are known. Their target point may be, among others, the cell membrane, cell wall, membrane ATPases, mitochondria and the endoplasmic reticulum. Furthermore, the expression of selected genes may be impaired, in particular, genes involved in adhesion, growth, dimorphism or sporulation, etc. [11]. Therefore, the next step in our research was to determine whether the antifungal activity of THY is related to a direct interaction with the cell wall structure and/or ion permeability of the *C. albicans* cell membrane.

Owing to its unique structure, the fungal cell wall represents a specific target for creating new antimycotics. It protects fungal cells against unfavorable changes in osmotic pressure and environmental stress factors while enabling contact with external conditions. In our study, the effect of THY on the fungal cell wall was assessed using sorbitol. Sorbitol, an osmotic protector, induces a mild cellular stress that can inhibit cell growth in the presence of selected cell wall blockers [11]. Therefore, the MIC of a cell wall damaging compound will be higher in the presence of an osmotic carrier [11,72]. The results of our study show that the MIC values ​​of THY in medium with and without sorbitol are the same. These findings are consistent with the absence of a major cell wall-targeting effect. The literature data only includes reports by Castro et al. [61], who performed similar studies. As with our results, their results show that the antifungal properties of THY probably are not related to the biosynthetic pathways of the cell wall because its values of MIC remained unchanged in the presence of sorbitol (MIC in presence of sorbitol was 39 μg/mL). According to Chatrath et al. [17], an osmotic stabilizer in the media could reverse the antifungal effect of the agents, which targets the fungal cell wall.

In our next study, we tested whether THY had an effect on ergosterol, the main component of the lipid bilayer cell membrane of fungi. This sterol, as well as various enzymes involved in its synthesis, are key targets for several classes of antimycotics used in the treatment of candidiasis. Among these drugs, the most important are polyenes and azoles [11,61,73]. Ergosterol is a structure specific to fungi, composed of unique fatty acids, that are found only in the cell membrane of fungi and are essential for their proper growth and functioning [19]. The substance is responsible for cell integrity [11,73] as well as for numerous cellular processes, including endocytosis, cell division, membrane fluidity, protein regulation in the membrane and cell signaling [11,71].

The effect of THY on the cell membrane was assessed using exogenous ergosterol. The basis of this study is the hypothesis that a compound that has an affinity for ergosterol rapidly forms complexes with free ergosterol in the medium, thus preventing interactions with ergosterol in the fungal membrane. As a result, the MIC of the tested compound increases [72,74]. The results indicate that MIC values of THY and NYS against *C. albicans* ​increase in medium supplemented with exogenous ergosterol as Compared to medium without sterol. Castro and co-workers [61] obtained similar results. These studies indicate that the MIC value of this phytocomponent towards *C. albicans* was 8-fold higher (from 39 μg/mL to 312.5 μg/mL) in an ergosterol environment.

Since polyene antibiotics act at the level of the fungal membrane [61], nystatin was used as a positive control. The above data may suggest that THY probably interacts with the plasma membrane, interferes with the biosynthesis and metabolism of ergosterol, increases the permeability of Ca2+ and K+ ions, proteins and radicals [11] and causes depletion of components necessary for the survival of fungal cells [61]. This leads, among others, to an increased concentration of reactive oxygen species (ROS) and oxidative stress, which causes a decrease of the extracellular polymer matrix (EPS) and capsular polysaccharide [19,40]. Additionally, it causes destabilization of the cell membrane, membrane-associated enzyme disturbances, and inhibition of fungal growth and ultimately leads to cell death [11,19,40]. Ahmad et al. [60] also hypothesized that THY enters the cell and blocks the ergosterol biosynthetic pathway, thereby impairing its biosynthesis. Moreover, some authors report that THY reacts with the membrane via its reactive hydroxyl group and that extensive membrane damage is the combined effect of multiple events [70].

The mechanism of action of THY may be related to its chemical structure, which gives it hydrophobic properties, which facilitates deposition on lipophilic elements of the fungal cell membrane. THY may also have an inhibitory effect on fungal proliferation, spore germination and cellular respiration [61].

### Influence of THY on *C. albicans* filamentation process

*C. albicans* as a dimorphic fungus can exist in the ovoid-shaped budding yeast form, which transitions to the branching filamentous hyphae form. Its invasiveness is contributed by the proficiency for phenotypic switching between yeast and filamentous forms and the ability to forming blastopores and chlamydospores [46]. In addition, hyphae, along with pseudohyphae and blastospores yeasts, are essential components of *C. albicans* biofilms [6]. Germ tube formation in *C. albicans* is one of the most important factors of pathogenicity, leading to the development of true hyphae. In turn, inhibition of this process may be a key therapeutic target due to the fact that hyphae show increased adhesion to host cells compared to planktonic yeast cells [35]. Therefore, we assessed the inhibitory impact of THY on the filamentation process at *C. albicans* strains. The obtained results show that THY, depending on its concentration, causes a gradual reduction in hyphal formation. A significantly beneficial effect was observed at a concentration of 1/2 MIC, where the filamentation was almost completely inhibited. Higher activity was observed at the MIC value resulting in the inhibition of yeast germination.

Research conducted by Jafri and Ahmad [58] reduced ability to form hyphae at *C. albicans* cells treated with THY was also observed. In addition, the authors indicated various morphological cell changes, namely, distorted shape and shrinkage of the cell membrane, as well as leakage of intracellular material. Abu Shaban and Darmani [35] demonstrated the inhibition of filamentation at a low-exposure THY concentrations (56% inhibition). These results indicate that under the influence of THY, well-formed, normal hyphae are transformed into distorted forms. Their complete absence was also confirmed. Other studies [35,68] reported that THY at a 2-fold higher inhibitory concentration strongly disturbs the formed biofilm due to significant damage to filamentous forms. These data highlight the importance of the tested natural compounds in modifying and inhibiting the production of germ hyphae at *C. albicans*. Their inhibition is very important because hyphae adhere to host cells much more strongly than planktonic cells. Moreover, the inhibition of yeast-to-mycelium transformation induced by THY is of great importance because this differentiation is essential for development, spread and infection. Yeasts colonize host surface sites by budding, while hyphae can effectively penetrate human cells and spread throughout the vascular system, causing systemic infections [35].

### Effect of THY on the expression of genes responsible for *C. albicans* adhesion and biofilm formation

Alterations in gene expression associated with biofilms have been proposed as a mechanism underlying the reduced susceptibility of *C. albicans* biofilms to antifungal agents [75]. In this study, the expression of eight genes implicated in adhesion, morphogenesis, metabolism, stress responses and biofilm regulation (*ALS3, HWP1, NRG1, TUP1, SAM2, CYS3, GRF10* and *BCR1*) was evaluated. RT-qPCR analysis was performed on ten clinical isolates identified as strong biofilm producers.

The RT-qPCR results are considered exploratory. Paired ΔCt values for biofilm-associated and planktonic cells are provided in Supplementary Tables S2 and S3, respectively. For biofilm-associated cells, *BCR1* exhibits the lowest unadjusted *p*-value, but does not reach statistical significance after Benjamini‒Hochberg correction for multiple comparisons. *ALS3* and *NRG1* also demonstrate non-significant directional changes, as did *HWP1*. No other gene met the adjusted significance threshold. In planktonic cells, no statistically significant changes were observed. Thus, none of the analyzed genes remained statistically significant after correction for multiple testing. The direction and magnitude of gene expression changes were determined using condition-specific median ΔCt values from Supplementary Table S2. *ALS3* exhibited the largest median-derived reduction in biofilm-associated cells, while *NRG1* showed the greatest median-derived increase. *BCR1* demonstrated a smaller median-derived increase but had the lowest unadjusted *p*-value. These findings indicate that the magnitude of the fold change does not necessarily reflect the strength of the statistical evidence, which also depends on the consistency of the responses among isolates. Within-condition analyses also do not reveal that the effect of THY differs between planktonic and biofilm-associated cells. Herein, a significant or suggestive result in one condition and a non-significant result in another does not, by itself, demonstrate a difference between conditions. As no direct test of interaction between treatment and growth state was performed, the results should be interpreted as separate accounts of responses in planktonic and biofilm-associated states.

Hierarchical clustering of log_2_(RQ) values reveals substantial variation among isolates in both the direction and magnitude of expression changes associated with THY (see Figure 6). Because RQ values were calculated relative to those of untreated control samples matched for experimental condition, the heatmaps reflect treatment-induced expression changes and do not provide information on basal gene expression levels. For visualization, the row and column dendrograms were cut at the highest bifurcation, resulting in two major groups for each dendrogram. The groups identified under biofilm-forming and planktonic conditions are descriptive and have not been validated as distinct biological response strategies. Therefore, the clustering results should be interpreted as evidence of response heterogeneity.

The biological functions of the analyzed genes provide context for the observed directional trends but do not elucidate the mechanism of THY action. *ALS3* and *HWP1* encode surface-associated proteins involved in adhesion, hyphal development and biofilm formation. If validated in larger datasets, a decrease in their expression would suggest reduced adhesion and filamentation. *NRG1* and *TUP1* are involved in the negative regulation of hyphal growth and the repression of hypha-specific genes [43–45]. Thus, increased expression of these genes would indicate enhanced repression of filamentation. However, these interpretations remain provisional, as the gene-specific changes are not statistically significant after correction for multiple testing.

*CYS3* is involved in the metabolism of sulfur-containing amino acids and maintenance of the cellular redox balance, while *SAM2* participates in the synthesis of S-adenosylmethionine, which is essential for methylation reactions, cellular metabolism and stress adaptation. Changes in the expression of these genes may indicate metabolic or stress-related responses to THY. *GRF10* functions as a transcription factor associated with adhesion and metabolic regulation, and *BCR1* serves as a key regulator of the biofilm gene network. Bcr1 and its downstream targets, including Als3 and Hwp1, contribute to adherence during biofilm formation [76]. The observed directional changes in these regulatory genes may reflect stress-related or compensatory responses. However, the current data do not demonstrate the activation of such compensatory pathways or the preservation of biofilm structure or viability.

Previous studies provide a biological context for these trends. Priya et al. [43] reported that exposure to *C. albicans* to sub-inhibitory concentrations of THY results in increased expression of the negative regulators *NRG1* and *TUP1* and decreased expression of genes associated with hyphal development. The directional effects observed in the present exploratory data are generally consistent with these findings, despite not reaching the adjusted significance threshold. Reduced expression of *HWP1* and *ALS3* has also been observed following exposure to other natural compounds. Linalool decreases the expression of both genes [77], while rutin, linalool, digitalin, atropine, epigallocatechin and lawsonide inhibit *ALS3* expression or its protein product [36]. In addition, cinnamaldehyde reduces *HWP1* expression and adhesion capacity [40], and sophorolipid lowers the transcript levels of both *HWP1* and *ALS3* [78].

Transcriptional responses of *C. albicans* biofilms to conventional antifungal agents have also been studied [75]. Accordingly, fluconazole does not significantly alter the expression of the biofilm-associated genes examined, whereas amphotericin B and caspofungin affect a broader range of transcripts. Caspofungin exposure is particularly associated with increased expression of hypha-specific genes, such as *ALS3* and *HWP1* [75], which contrasts with the non-significant downward changes observed for these genes in the present study. However, such comparisons should be interpreted with caution owing to differences in compounds, concentrations, exposure conditions, analytical methods and biological models across studies.

Several limitations should be considered in interpreting these findings. First, in our work, biofilm quantification relied on crystal violet staining, which measures total biomass but does not distinguish between viable and non-viable cells or extracellular matrix material. Second, RT-qPCR analysis included only 10 isolates that were all strong biofilm producers. This small, phenotypically limited sample may have resulted in low statistical power and may not represent the full range of variability among clinical isolates. Third, gene expression was assessed at a single THY concentration, precluding evaluation of concentration-dependent transcriptional changes. Fourth, normalization relied on a single reference gene, *UBC13*. Its expression stability was not formally evaluated either between untreated and THY-treated samples within each growth state or across planktonic and biofilm-forming conditions. Potential variation in *UBC13* expression may therefore have introduced bias into the estimated relative-expression changes. The RT-qPCR findings should consequently be regarded as exploratory and require confirmation using multiple reference genes empirically validated under relevant experimental conditions. Finally, eight genes were examined per growth condition, and none remained statistically significant after correction for multiple comparisons; therefore, the gene expression and clustering results should be regarded as exploratory and hypothesis-generating.

Taken together, the results demonstrate that THY exhibits fungicidal and antibiofilm activity against oral *C. albicans* isolates. These results support a membrane-associated effect with the inhibition of filamentation. RT-qPCR data further indicate heterogeneous, isolate-dependent directional changes in genes involved in adhesion, morphogenesis, stress adaptation and biofilm regulation. However, these transcriptional trends do not establish coordinated modulation of the biofilm regulatory network or the involvement of a specific molecular mechanism.

## Conclusions

THY demonstrates moderate to strong antifungal activity and exhibits a fungicidal effect against oral *Candida albicans* isolates collected from hospital patients with hematological malignancies. It inhibits biofilm formation and reduces the biomass of established biofilms in a concentration-dependent manner. However, higher concentrations are generally required to impact mature biofilms. These findings reflect alterations in total biofilm biomass. The observation that MIC values remain unchanged in the presence of sorbitol suggests that THY does not primarily target the cell wall. In contrast, the 8- to 16-fold increase in the MIC observed with the addition of exogenous ergosterol may indicate a mechanism associated with the cell membrane or dependent on ergosterol. Additionally, THY inhibits *C. albicans* filamentation in a concentration-dependent manner. These findings showed that THY probably acts through membrane-associated mechanisms and inhibits key virulence-related trait, although a specific molecular target has yet to be identified.

The RT-qPCR results are exploratory in nature. For biofilm-associated cells, *BCR1* exhibits the lowest unadjusted *p*-value. However, statistical significance was not achieved after applying the Benjamini–Hochberg correction. Similarly, the directional changes observed for *ALS3, HWP1* and *NRG1* did not reach statistical significance following adjustment, and no significant gene expression changes were detected under planktonic conditions. Consequently, none of the eight genes analyzed met the adjusted significance threshold, and the observed expression changes should be considered hypothesis-generating.

In summary, these results support continued investigations of THY as a potential antifungal and antibiofilm agent. To elucidate its mechanism of action and therapeutic potential, future studies should use larger, more phenotypically diverse collections of isolates, incorporate validated reference-gene normalization, assess a range of THY concentrations, employ viability-based bioassays and apply appropriate experimental approaches to evaluate both efficacy and safety.

## Supplementary Material

Supplementary material Supplemental Material

Supplementary material Supplemental Material

Supplementary material Supplementary_Table_S3-ZJOM-S-2026-0085.R3.docx

## Data Availability

The data will be available upon request to the corresponding author.
