# Supplementary material for: Mechanism of anti-biofilm thymol activity in oral Candida albicans strains – phenotypic and genotypic level
Source: J Oral Microbiol. 2026 Oct 29;18(1):2741862. doi: 10.1080/20002297.2026.2741862 (PMC13721810; doi:10.1080/20002297.2026.2741862)
Supplement: Supplementary file 1 [file ZJOM_A_2741862_SM4795.docx]

Supplementary Table S1. The strains used for RT-qPCR.

| Tested isolates | MIC  (μg/mL) | MFC  (μg/mL) | ≥50% inhibition  of biofilm  (μg/mL) | ≥50% reduction  of biofilm  (μg/mL) |
| --- | --- | --- | --- | --- |
| 20 | 125 | 250 | 250 | 500 |
| 128 | 500 | 1000 | 500 | 4000 |
| 64 | 250 | 500 | 500 | 2000 |
| 63 | 250 | 250 | 250 | 1000 |
| 47 | 250 | 500 | 500 | 2000 |
| 21 | 125 | 500 | 250 | 1000 |
| 25 | 500 | 500 | 500 | 2000 |
| 19 | 250 | 250 | 500 | 1000 |
| 74 | 500 | 1000 | 2000 | 4000 |
| 33 | 250 | 500 | 1000 | 2000 |
