# Supplementary material for: Mechanism of anti-biofilm thymol activity in oral Candida albicans strains – phenotypic and genotypic level
Source: J Oral Microbiol. 2026 Oct 29;18(1):2741862. doi: 10.1080/20002297.2026.2741862 (PMC13721810; doi:10.1080/20002297.2026.2741862)
Supplement: Supplementary file 2 [file ZJOM_A_2741862_SM4809.docx]

Supplementary Table S2. Statistical analysis of gene expression changes induced by THY in biofilm-associated *C. albicans* cells. For each gene, median ΔCt, mean ΔCt ± SD, and p-values obtained using the Wilcoxon matched-pairs signed-rank test are presented. Technical triplicates were averaged before statistical analysis. A *q*-value ≤ 0.05 was considered statistically significant.

| Gene | Condition | Median ΔCt | Mean ΔCt | ±SD | Raw *p* | BH-adjusted q |
| --- | --- | --- | --- | --- | --- | --- |
| *ALS3* | untreated | -2.751 | -2.562 | 2.422 |  |  |
| *ALS3* | treated | -0.653 | -1.415 | 2.107 | 0.065 | 0.141 |
| *CYS3* | untreated | -3.367 | -3.298 | 0.928 |  |  |
| *CYS3* | treated | -3.916 | -3.776 | 0.680 | 0.106 | 0.141 |
| *HWP1* | untreated | -2.879 | -3.160 | 2.858 |  |  |
| *HWP1* | treated | -1.355 | -1.800 | 3.111 | 0.084 | 0.141 |
| *NRG1* | untreated | 4.572 | 4.065 | 3.209 |  |  |
| *NRG1* | treated | 2.321 | 2.469 | 1.936 | 0.065 | 0.141 |
| *SAM2* | untreated | -2.950 | -3.456 | 1.214 |  |  |
| *SAM2* | treated | -3.085 | -3.111 | 1.102 | 0.432 | 0.432 |
| *TUP1* | untreated | -1.952 | -1.944 | 0.524 |  |  |
| *TUP1* | treated | -2.015 | -2.196 | 0.859 | 0.322 | 0.368 |
| *GRF10* | untreated | 0.531 | 0.679 | 1.444 |  |  |
| *GRF10* | treated | 0.082 | 0.223 | 1.700 | 0.106 | 0.141 |
| *BCR1* | untreated | 0.265 | 0.537 | 2.611 |  |  |
| *BCR1* | treated | -1.243 | -0.546 | 3.267 | 0.010 | 0.080 |
