# Supplementary material for: Mechanism of anti-biofilm thymol activity in oral Candida albicans strains – phenotypic and genotypic level
Source: J Oral Microbiol. 2026 Oct 29;18(1):2741862. doi: 10.1080/20002297.2026.2741862 (PMC13721810; doi:10.1080/20002297.2026.2741862)
Supplement: Supplementary file 3 [file ZJOM_A_2741862_SM4817.docx]

Supplementary Table S3. Statistical analysis of gene expression changes induced by THY in planktonic *C. albicans* cells. For each gene, median ΔCt, mean ΔCt ± SD, and p-values obtained using the Wilcoxon matched-pairs signed-rank test are presented. Technical triplicates were averaged before statistical analysis. A *q*-value < 0.05 was considered statistically significant.

| Gene | Condition | Median ΔCt | Mean ΔCt | ±SD | Raw *p* | BH adjusted q |
| --- | --- | --- | --- | --- | --- | --- |
| *ALS3* | untreated | -3.113 | -3.133 | 1.626 |  |  |
| *ALS3* | treated | -2.224 | -1.848 | 1.865 | 0.193 | 0.770 |
| *CYS3* | untreated | -2.472 | -2.289 | 1.106 |  |  |
| *CYS3* | treated | -2.064 | -2.002 | 0.969 | 0.492 | 0.770 |
| *HWP1* | untreated | -3.019 | -3.366 | 2.323 |  |  |
| *HWP1* | treated | -2.927 | -2.256 | 2.982 | 0.322 | 0.770 |
| *NRG1* | untreated | 5.845 | 5.232 | 3.180 |  |  |
| *NRG1* | treated | 4.665 | 4.720 | 3.604 | 0.625 | 0.770 |
| *SAM2* | untreated | -4.867 | -4.749 | 0.784 |  |  |
| *SAM2* | treated | -4.405 | -4.315 | 0.698 | 0.193 | 0.770 |
| *TUP1* | untreated | -2.606 | -2.712 | 0.669 |  |  |
| *TUP1* | treated | -2.441 | -2.610 | 1.155 | 0.770 | 0.770 |
| *GRF10* | untreated | 1.228 | 1.108 | 1.704 |  |  |
| *GRF10* | treated | 1.292 | 1.137 | 1.553 | 0.695 | 0.770 |
| *BCR1* | untreated | -0.406 | -0.206 | 1.552 |  |  |
| *BCR1* | treated | -0.887 | -0.018 | 3.033 | 0.625 | 0.770 |
